# When and how do hospital nurses cope with daily stressors? A multilevel study

**DOI:** 10.1371/journal.pone.0240725

**Published:** 2020-11-10

**Authors:** Fermín Martínez-Zaragoza, Gemma Benavides-Gil, Tatiana Rovira, Beatriz Martín-del-Río, Silvia Edo, Rosa García-Sierra, Ángel Solanes-Puchol, Jordi Fernández-Castro

**Affiliations:** 1 Department of Behavioural Sciences and Health, University Miguel Hernández, Elche, Spain; 2 Departament de Psicologia Bàsica, Evolutiva i de l'Educació, Universitat Autònoma de Barcelona, Barcelona, Spain; 3 Department of Nursing, Universitat Autònoma de Barcelona, Bellaterra, Cerdanyola (Barcelona), Spain; Universitat de Valencia, SPAIN

## Abstract

**Background:**

During their workday, nurses face a variety of stressors that are dealt with using different coping strategies. One criticism of the contextual models of work stress is that they fail to focus on individual responses like coping with stress. Neverthless, little is know about the momentary determinants of coping in nurses.

**Objectives:**

To identify the momentary predictors of problem-focused approaching coping and emotion-focused approaching coping, as well as those for seeking social support and refusal coping strategies, during the working day in nurses.

**Design:**

This study uses descriptive, correlational, two-level design with repeated measures.

**Settings:**

Wards of two University hospitals.

**Participants:**

A random cohort of 113 nurses was studied.

**Methods:**

An ecological momentary assessment was made of demand, control, effort, reward, nursing task, coping, mood and fatigue, and of coping style by questionnaire. Multilevel two-level statistical analyses were performed in order to identify both within person and between person relationships.

**Results:**

Different momentary types of coping were associated with different tasks. The problem-focused coping could be explained by the direct care and medication tasks, demand, planning coping style, mood, and negatively by acceptation coping style. Emotion-focused coping could be explained by documentation and medication tasks (negatively), mood, demand, distraction, and disengagement coping styles. Seeking social support coping could be explained by the task of communication, mood, fatigue (negatively), and seeking emotional support as a coping style. Refusal coping could be explained by mood, and the coping style of focusing and venting emotions. Refusal coping is not specific to any task.

**Conclusions:**

The choice of the coping strategy depends on the task, of their appraisal and on the different styles of coping.

## Introduction

Nurses are exposed to different risk factors, such as the organizational climate, irregular hours, excessive workloads, insufficient support from supervisors and partners, violence at work, and contact with suffering or death [1–5]. These factors may have important consequences for the physical and mental health of these professionals, influencing their performance at work and provoking hypertension, asthma, loss of concentration, apathy, loss of motivation, difficulties in decision making, reduced efficacy, depression, insomnia, anxiety, etc. [6, 7].

There are several studies indicating that nurses show moderate levels of fatigue and poor recovery between shifts, which can increase the risk of significant failures in attention, a deterioration in performance, errors and accidents, all of which may affect the safety of both staff and patients [8–11].

Moreover, nurses who suffer strong psychological demands in conjunction with a lack of control over their work appear to be at a higher risk of developing physical and psychological problems [12, 13]; this combination of factors is well established in the Demand/Control Work Stress Model [14–16]. The development of chronic stress in a work context has also been explained by the Effort-Reward Imbalance Model [17]. This model states that work stress occurs due to an imbalance between the effort made by the worker and the reward received for this effort. Thus, in the short- to mid-term an imbalance between effort and reward would generate greater vulnerability to disease, e.g., gastrointestinal and musculoskeletal disorders, hypertension or cardiovascular disease, and psychological problems [18, 19]. Among nurses, such an imbalance seems to be associated with frequent short episodes of sick leave, an intention to leave their job, job turnover, work-home conflicts, burn-out, emotional exhaustion, low perceived health, depression and anxiety [20–23].

One criticism of the contextual models discussed above is that they fail to focus on individual responses like coping with stress (i.e. [24]). The transactional model of stress considers coping as a process that varies according to the demands of a given situation [25], giving more importance to how an individual reacts to a particular stressor than to other dispositional variables [26]. It is even suggested that coping might be more important than the level of stress itself [27–29]. Studies carried out internationally show that the coping strategies preferred by nurses in the workplace are planned problem-solving, self-control, seeking social support and positive reappraisal [30–35] and several studies have highlighted the influence of the type of coping strategies used by nurses on their level of stress, as well as on their health status [6, 36, 37], concluding that nurses who use ineffective coping strategies are at greater risk of experiencing stress, as well as a variety of physical and psychological pathologies [37, 38]. Indeed, problem-focused coping is used more often than emotion-focused coping to manage stressful work situations associated with nursing, contributing positively to reducing stress, as well as improving performance and job satisfaction [39–41].

However, it is not always true that adaptive coping focuses on the problem and maladaptive coping on emotion [42, 43]. Likewise, several studies carried out with intensive care, palliative care and emergency nurses showed that emotion-focused coping strategies are used to a large extent in these units, such as positive thinking, religious beliefs or spirituality [6, 44]. Therefore, to understand how coping strategies are related to better adjustment, a problem-approaching/emotion-approaching/refusal classification should be contemplated. Some problem-focused strategies indicate that individuals are facing up to the problem (e.g. problem solving), whereas others are the consequence of a refusal to face it (e.g. avoidance). Some emotion-focused strategies imply facing up to the problem (e.g. positive reinterpretation) while others imply refusal (e.g. self-blame: [45]). Although research indicates that no coping strategy works well for all individuals and/or situations [28, 46], and that better results can be obtained by combining several strategies [28, 47], approaching strategies are generally more closely related to a better psychological adjustment that refusal ones. Moreover, another group of coping strategies especially important when adjustment is at stake is seeking social support [48]. Social support can help cope with the emotional demands of a situation (e.g. to get sympathy and understanding from someone) or to solve problems (e.g. getting advice).

Finally, as Skinner, Edge, Altman, and Sherwood (2003) [49] have pointed out, people do not face stress by choosing between options incompatible with each other but that attempts to cope with stress fulfil different functions simultaneously such as coordinating actions, conserving resources and adjusting expectations. For all this, it is crucial to assess the coping at the time it occurs through categories that are not exclusive.

Some studies have determined that reports of momentary coping are not necessarily consistent with more general styles of coping employed [50]. Similarly, they have addressed whether the variation found is better explained by context-momentary variables or by dispositional ones, modelling the between-person and within-person effects all in one. In nurses, it appears that negative mood is greater in moments of high demand/effort with low control/reward, and that a high positive mood is related to moments of high task demand/effort and high control/reward [51]. In another study [52], emotional exhaustion was found to make all work tasks less rewarding, and it was related to a more negative mood and greater fatigue. However, to the best of our knowledge, what determines the type of coping chosen at a given time by nurses has yet to be investigated.

Thus, the aim of this study was to identify the momentary predictors of problem-focused approaching coping and emotion-focused approaching coping, as well as those for seeking social support (instrumental or emotional) and refusal coping (emotion or problem-focused) strategies, during the working day in nurses. To achieve this goal it will be necessary to use the method of the ecological momentary assessment [45, 53, 54] to evaluate the task carried out at each moment as well as the demand, effort, reward and control appraised in the moment, the mood and fatigue at that moment and coping. It is important to note the type of nursing task performed because the work stress vary in function of whether they are carrying out direct care and medication or any other task. Social dimension of the task is another essential distinction, i.e. ward meetings, tutoring, and professional communication versus individual ones. To better understand the influence of momentary variables on momentary coping, the style of coping was also considered in order to control its influence on momentary coping-strategies. As such, and based on the current state-of-the-art, we hypothesized that:

- Problem-focused coping approaches will be chosen by nurses in direct care or medication tasks with high demands and requiring much effort.
- Emotion-focused coping approaches will not be related to the type of task, although this kind of coping will be expected in conditions of high demands and effort, little control, negative mood and high states of fatigue.
- Seeking-support coping will be chosen in tasks that involve other nurses, probably tasks with high demands and effort, little control, negative mood and high states of fatigue.
- Refusal coping will not depend on the type of task, and it is expected to be employed in tasks of high demand and effort, little control and reward, and in relation to negative mood and high states of fatigue.

## Methods

Approval of the study was granted by CEIC (Ethics and Clinical Research Committee) at the University Hospital of Elche, Spain, and the CEIC at the Hospital of Terrassa, Spain. Each participants signed an informed consent.

This paper is the second to be prepared from a research project assessing stress in nurses, the first of which focused on the effect of emotional exhaustion on nurses [52]. Although the present article shares the methodology with the former, its objective differs completely, focusing specifically on coping.

### Participants

A random cohort of 113 nurses was recruited from the following wards at two University hospitals in Spain: internal medicine, surgery, traumatology, oncology, cardiology, neurology, nephrology, pneumology, rheumatology, digestive, gynaecology, geriatrics, palliative care, paediatrics, and psychiatry. Critical care services and emergency services were excluded from the sample because of their distinctive features. The data was collected individually between January and December of 2015, excluding holidays periods. Every nurse was cited to explain him/her the procedure and how to complete the data. The nurse-patient ratio at these hospitals ranges from one nurse per 10 patients on the day shift or per 30 patients on the night shift. Of the nurses invited to participate, 17 refused such that the final cohort was comprised of 96 nurses, with a response rate of 84.95%.

### Instruments

The data collected was structured in two levels: Level 1 was within-subject and consisted of taking repeated measurements over time for each participant, and Level 2 was between-subjects and consisted of applying a set of questionnaires only once to each subject.

### Level 1 measures (moment): Ecological momentary assessment

Measurements were obtained using a Samsung Galaxy Mini Smartphone with Android software specially developed for this study. Data entry was prompted by vibration or a buzzing alarm and if busy, the nurse could postpone the response for 10 minutes. This meant that if the task they were involved in was direct care, it could be completed and the nurses could wash their hands before touching the screen. However, if the question remained unanswered for 20 min then this moment was registered as missing data. Answers were presented on analogue scales and they were given ‘tips' to select their responses to the questions. The software was designed with the help of a menu to answer any queries by just touching the screen. The measurements taken at each evaluation point are listed below.

#### Mood

**Fig 1:**
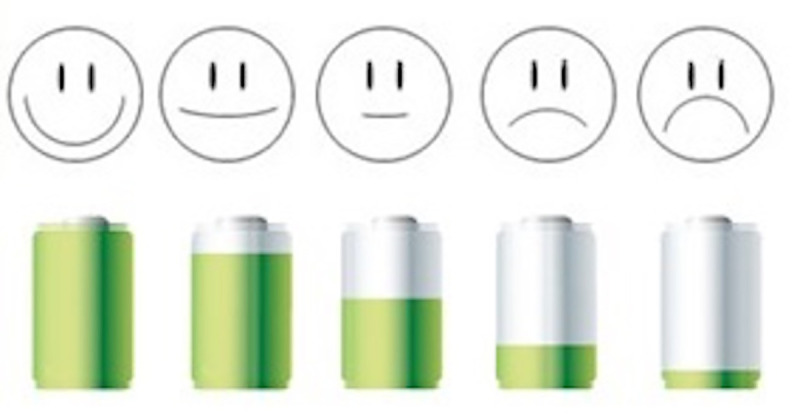
Analogue scales of mood and fatigue.

Mood was measured on a single-item, visual analogue scale of five points, from a happy face to a sad face, where high values of mood reflect negative mood (see Fig 1).

#### Fatigue

Fatigue was measured by a single-item, visual analogue scale of five points, from a full battery to an empty battery (see Fig 1), where high values of fatigue mean high levels of fatigue.

#### Nursing task

The task the nurse was involved in was coded according to an adaptation [52] of the WOMBAT classification [55] and classified as: direct care, indirect care, medication, documentation, communication, and social/resting tasks.

#### Demand, effort, control, reward

Four questions were designed to appraise the different characteristics of work stress: demand, control (labelled as autonomy and skills development), effort, and reward. Each question labelled the term to be evaluated and was followed by a simple question as to how far each concept could be applied to the characteristics of the task performed at the time. The four questions were answered on a visual analogue scale from 0 to 10 in order to evaluate the intensity of the response, one of the most usual response formats for single-item questions [56].

#### Momentary coping

A 10-item coping questionnaire was designed ad-hoc to assess nursing coping in an ecological momentary assessment context (*MoCoping*: [57]), based on the COPE Inventory [58, 59]. The questionnaire follows a structure of one item for each strategy, grouped into four types: a problem-focused coping approach (including one item of active coping and one of planning); an emotion-focused coping approach (including one item of acceptance, one of reinterpretation and one of distraction); seeking social support coping (including one item of emotional support and one of instrumental support); and refusal coping (including one item of denial, one of venting and one of self-blame). The structure of the questionnaire was agreed by three experts, including the four relevant types of coping and their relevant strategies, also proposing an initial 10-item selection. To assess the construct validity, we asked 85 nursing students to answer the COPE and another coping questionnaire related to their hospital practice (CRI-A: [60]). The initial items proposal were then reviewed according to three criteria: best loading factor, greatest decrease in the alpha index when deleted, and the applicability to an ecological momentary assessment nursing context. The 10 items proposed were then correlated with the corresponding CRI-A strategy to test its representativeness. A final pilot study was carried out on 5 nurses to test the feasibility, answering the MoCoping questionnaire following the same procedure described here. Nurses were asked to indicate which coping strategies they used to handle difficulties that might have arisen during an activity or task, or as a consequence of it. The final options include: relax/disconnect (distraction coping); expressing discomfort to other people (venting); try to get advice/help from others on what to do (seeking instrumental support); performing a direct action for resolution (problem solving); pretending it does not affect me (denial); talking with someone about how I feel (seeking emotional support); accepting the situation (acceptance); thinking of a solution (planning); looking for some good in the situation (reinterpretation); criticizing/blaming myself (self-blame); or none of these. The four types of coping were computed on a binary scale, with 1 representing at least one strategy of the corresponding type selected. The results showed that the MoCoping scale was feasible, as nurses reported variability in the frequency and type of coping used at different points in time, and the pattern of strategies used was similar to those employed in retrospective assessments, albeit with an increase of emotional strategies, as found in other studies that assessed coping in other ecological momentary assessment contexts [50].

The order of each record at a person level (level 1, moment) was automatically recorded by the device, registering a mean of five records per shift.

### Level 2 measures (person): Questionnaires

#### Ad-hoc questionnaire

We recorded the gender, age, marital status, number of children, years of experience and professional status of the subjects.

#### Coping style

The COPE Inventory was used in this work ([58]; Spanish adaptation by Crespo and Cruzado, 1997 [59]), including the scales equivalent to those measured on the momentary coping scale (see below): Active coping (5, 25, 47, 58), Planning (19, 32, 39, 56), Seeking of instrumental social support (4, 14, 30, 45), Seeking of emotional social support (11, 23, 34, 52), Focusing and venting of emotions (3, 17, 28, 46), Acceptance (13, 21, 44, 54), Denial (6, 27, 40, 57), Positive reinterpretation (29, 38) and Distraction (2, 43). As the momentary coping scale also includes the self-blame strategy (refusal coping) that is not included in the COPE Inventory, the scale was selected from the brief COPE Inventory (Items 13, 26; [61]). Therefore, the final inventory consisted of 34 items evaluated using a four-point Likert scale: 0 = not at all; 1 = a little bit; 2 = quite; 3 = a lot.

### Procedure

Nurses were recruited from a list provided by the Human Resources Departments of all the nurses working on the hospital wards, maintaining their anonymity. The inclusion criteria required nurses to have an ongoing full-time contract, such that nurses who were not currently working were excluded. A random sample of 80% of the ward nurses at each hospital was selected and the nurses were asked to participate voluntarily. A member of the research team explained the purpose of the study and its execution to each nurse individually, giving them written instructions. After agreeing to participate, the nurses provided their signed informed consent, basic demographic and professional details were collected, and they were provided a sealed envelope with the set of questionnaires prepared for this research. They were also provided with a smartphone programmed to schedule their next five shifts and they were shown how the smartphone worked, making sure they had clearly understood how to use it. They were also given a contact phone number to report their completion of the evaluation or to address any mishap that may have occurred. Research assistants collected both the completed questionnaires and the smartphones after the procedure was completed. The data were collected over a period of six months.

Nurses participating in the study signed an informed consent, being able to leave the study at any moment should they desire. In addition, approval for the study was granted by the ethics and clinical research committees of the participating hospitals (no code numbers), and the study was carried out in compliance with the Helsinki declaration regarding research on Humans.

### Data analyses

Multilevel statistical analyses were performed in order to identify both within person and between person relationships. This study uses a two-level design with repeated measures [62] in which the level 1 is established from the moment the outcome variables measured were taken, and in level 2, these moments are nested on the person level. No missing data imputation was done.

#### Multilevel modelling

Multilevel analysis allows the variance associated to random factors to be controlled without data aggregation. As fixed effects, we entered the four different types of momentary coping strategies into the model, as well as the coping styles, the nursing tasks, demand-control and effort-reward, as well as mood and fatigue. As random effects, we tested the random intercepts and slopes for the effect of the same variables, allowing them to vary randomly across the groups. P-values were obtained as likelihood ratios of the full model with each effect against the model without the effect. Z-values were obtained to test the significance of fixed effects, the estimates and standard errors in the tables.

#### Assumptions

Visual inspection of the q-q plots with the car R package [63] did not reveal any obvious deviations from homoscedasticity or normality in the dependent variables. The four momentary types of coping and the different type of tasks were categorized and coded as dummy variables, such that logit models were used to predict the type of coping. The rest of the variables were quantitative.

#### Initial models, model fit and fit criteria

The model comparison approach followed the guidelines of Bliese and Ployhart (2002) [64] and Bliese (2016) [65], beginning the process by examining the nature of the outcome. To test the significance of the person effects, we carried out a likelihood ratio test to compare the null multilevel model (unconditional model) with a null single-level model, thereby testing the null hypothesis that there are no group differences. Subsequently, the intraclass correlation coefficient was estimated to calculate the between/within variation ratio. The intraclass correlation coefficient helps determine whether or not a linear mixed model is necessary, and it is also meaningful to see how the intraclass correlation coefficient changes as variables are added to the model [66]. Finally, level 1 and level 2 predictors of the intercept and slope variances were added from the simplest model, giving more complex models (unconditional growth models) as recommended by Hox (1995) [67]. Random slope models and cross-level interactions were finally tested (conditional growth models). The model fit was assessed using chi-squared tests of the log-likelihood values to compare different models and by using the Akaike's information criterion (AIC: [68]), a relative goodness of fit index. According to the change in these indices, the model with the last significant change was chosen for each analysis.

The predictors tested for the use of momentary coping strategies were: 1) nursing tasks, 2) demand and control, 3) effort and reward, 4) mood and fatigue, and 5) coping styles. The data was analysed with the R Statistical Package [69], using the lme4 R package [70] to analyse the binary variables (logit models, glmer procedure), a hierarchical mixed effects, univariate, two-level regression model with repeated measures analysis was generated, and a random intercept and slope (multilevel longitudinal growth curve model) analysis was performed on the relationship between momentary coping, coping style, tasks, evaluation (demand, control, effort, and reward), and mood and fatigue. P-values of the lme4 outputs were obtained using the lmer Test package [71]. Different measures were considered: problem-focused coping, emotion-focused coping, seeking social support coping and refusal coping approach; direct-care, indirect-care, documentation, medication, social and communication tasks; demand, control, effort and reward; mood and fatigue; and coping styles, such as seeking instrumental social support, seeking emotional social support, problem-solving, planning, focusing and venting emotions, acceptance, negation, positive reinterpretation, self-blame and distraction.

The intraclass correlation coefficient was calculated using the sjstats R package [72] and the graphical data was processed with the ggplot2 R software package [73].

## Results

### Characteristics of study population

In the cohort analysed, 89.90% of the subjects were female with a mean age of 40.22 years (SD = 8.50), distributed 47.42/52.58% between the two hospitals. Regarding the shifts they worked 45.45% were on rotating shifts, while the remaining 54.55% working fixed shifts (either mornings, evenings or nights), with 5.20% maintaining an additional job. The mean time spent in their current job was 9.86 (SD = 7.99) and their mean experience as a nurse was 17.40 years (SD = 8.36). Indeed, 77.31% of the nurses were tenured staff and 52.53% had specialist training in nursing over and above their university degree. In addition, 50.51% were in a relationship, 16.16% were single and the remaining subjects were either separated, divorced or widowed.

### Coping strategies

A problem-focused coping strategy was used in 45.25% of the moments measured, an emotion-focused coping in 44% of the moments, with seeking social support coping employed in 8% and refusal coping in 2.75%.

**Fig 2:**
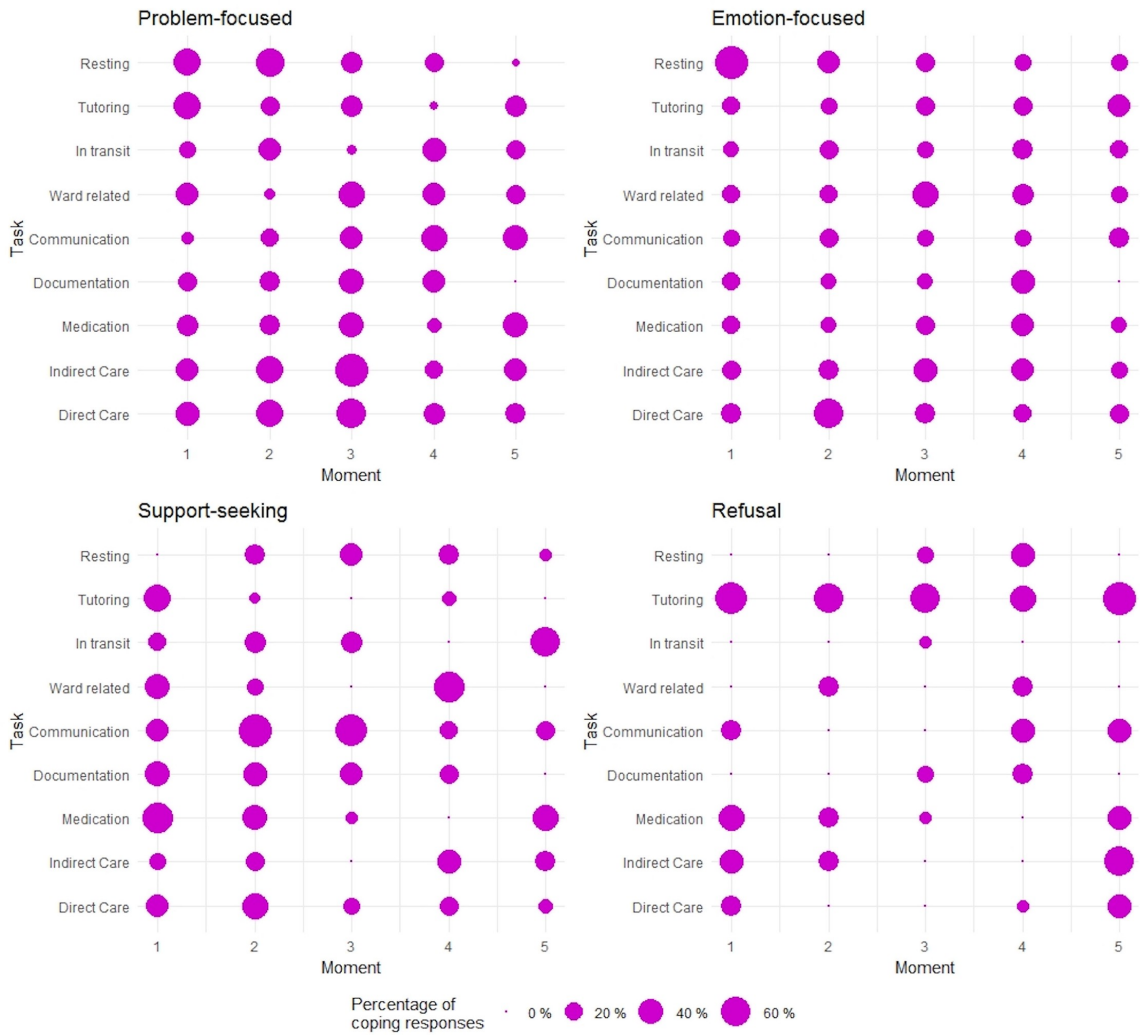
Frequencies of the different types of coping strategies depending on the task performed and the time point during the shift.

The frequencies of the different types of coping were assessed in relation to the tasks performed and the time point in the shift that the measure was registered (Fig 2). A problem-focused coping approach was particularly frequent during the first three time points in the shift, and higher values were evident in association with tutoring, direct care and indirect care. By contrast, the frequencies of emotion-focused coping approaches were distributed more widely, whereas seeking social support coping was most often seen in relation to medication, tutoring and communication, and mainly in the first three time points in the shift. Refusal coping approaches were most frequent in the tutoring task, independent of the time during the shift.

The intraclass correlation coefficient of the final model fitted to the different coping strategies indicated that: 61.60% of the variation for problem-focused coping was found at level 1 (moments) and 38.40% at level 2 (within person); for emotion-focused coping 57.02% of the variation was at level 1 (between moments) and 42.98% at level 2, for seeking social support coping 69.33% of the variation was found at level 1 (moments) and 30.67% at level 2 (within person); while for refusal coping, 81.59% of the variation was found at level 1 (moments) and 12.41% at level 2. The multilevel analysis (binary, multilevel logit model; see Tables 1 and 2) showed model 5 to be the best fit for problem-focused coping, a model of a random intercept type and thus, it assumed that the participants had different initial values. By contrast, model 6 proved to be the best fit for emotion-focused coping, seeking social support coping and refusal coping (Table 2), again a random intercept and slope type model that assumed that the participants had different initial values and followed different slopes.

**Table 1:** Fixed effect (t-test, top) and variance estimates (standard deviation, bottom), and the indices of fitness for models predicting problem-focused (PFC) and emotion-focused approaches to coping (EFC).

Parameter	Model 1: Null multilevel model		Model 2: + tasks		Model 3: + D/C, E/R		Model 4: + coping style		Model 5: + modo + fatigue		Model 6:+ random slope	
Moment. coping	PFC	EFC	PFC	EFC	PFC	EFC	PFC	EFC	PFC	EFC	PFC	EFC
**Fixed effects**												
Intercept	-1.36 (0.25)***	-1.43 (0.23)***	-1.89 (0.27)***	-1.26 (0.24)***	-3.55 (0.34)***	-1.67 (0.27)***	-4.74 (0.95)***	-2.19 (0.37)***	-5.41 (0.95)***	-2.67 (0.40)***		-3.02 (0.44)***
**Level 1 (Moment)**												
Tasks:												
DirCare			1.16(0.16)***		0.48(0.17)**		0.49(0.18)**		0.42(0.18)*			
Medic			0.73(0.18)***	-0.58(0.17)**	0.45(0.19)*	-0.56(0.17)***	0.46(0.19)*	-0.56(0.17)***	0.48(0.20)*	-0.53(0.17)**		-0.53(0.17)**
Docum				-0.48(0.18)**		-0.48(0.18)**		-0.48(0.18)**		-0.46(0.18)*		-0.45(0.18)*
Demand					0.34(0.03)***	0.08(0.02)**	0.33(0.03)***	0.08(0.02)**	0.31(0.03)***	0.05(0.02)*		0.06(0.02)*
Mood									0.31(0.08)***	0.26(0.07)***		0.40(0.09)***
**Level 2 (Person)**												
Coping styles:												
Planif							0.38(0.11)**		0.39(0.11)***			
Accep							-0.26(0.10)*		-0.26(0.10)*			
Diseng								0.51(0.24)*		0.51(0.24)*		0.46(0.21)*
**Random effects**												
**Level 1 (Moment)**												
Intercept SD	2.28	2.14	2.37	2.15	2.46	2.10	2.34	2.06	2.29	2.05		2.95
**ICC and Fitness indices**												
ICC	.61	.58	.63	.58	.64	.57	.62	.56	.61	.56		.72
AIC	1662	1739	1612	1730	1514	1723	1506	1721	1495	1712		1705
BIC	1673	1750	1634	1752	1541	1751	1544	1754	1539	1751		1755
Sig. of fit change (ANOVA)	***	***	***	***	***	**	**	*	***	***	Failed to converge	**

Note: Standard errors are in parenthesis

*p < .05

**p < .01

***p < .001.

Momentary coping: PFC, Problem-focused approach; EFC, Emotion-focused approach. Tasks: DirCare, direct care; Medic, medication; Docum, documentation. Coping styles: Planif, planification; Accep, acceptance; Diseng, disengagement. ICC, Intraclass Correlation Coefficient; AIC, Akaike Information Criterion; BIC, Bayesian Information Criterion.

**Table 2:** Fixed effect (t-test, top) and variance estimates (standard deviation, bottom), and the fitness indices for models predicting support-seeking (SSC) and refusal coping (RC).

Parameter	Model 1: Null multilevel model		Model 2: + tasks		Model 3: + D/C, E/R		Model 4: + coping style		Model 5:+ mood + fatigue		Model 6: + random slope	
Moment. coping	SSC	RC	SSC	RC	SSC	RC	SSC	RC	SSC	RC	SSC	RC
**Fixed effects**												
Intercept	-3.31 (0.23)***		-3.45 (0.24)***			-4.81 (0.66)***	-4.48 (0.59)***	-5.81 (0.82)***	-4.96 (0.67)***		-6.07 (0.77)***	-9.74 (1.32)***
**Level 1 (Moment)**												
Tasks:												
Com			1.32(0.30)***				1.31(0.30)***		1.34(0.31)***		1.33(0.31)***	
Demand						0.16(0.06)*		0.14(0.06)*				
Reward						-0.23(0.07)**		-0.22(0.07)**				
Mood									0.55(0.12)***		0.91(0.16)***	1.20(0.33)***
Fatigue									-0.40(0.13)**		-0.38(0.12)**	
**Level 2 (Person)**												
Coping styles:												
Emot							0.14(0.07)*				0.17(0.06)**	
Focus								0.27(0.10)**	0.16(0.07)*			0.28(0.10)**
**Random effects**												
**Level 1 (Moment)**												
Intercept SD	1.45		1.47			1.86	1.45	1.65	1.47		2.72	3.94
**ICC and Fitness indices**												
ICC	.39		.39			.51	.39	.45	.39		.69	.82
AIC	899		885			379	882	374	867		858	356
BIC	910		901			401	904	402	900		903	389
Sig. of fit change (ANOVA)	***	Failed to converge	***	Failed to converge	n.s.	***	*	**	***	n.s.	**	***

Note: Standard errors are in parenthesis

*p < .05

**p < .01

***p < .001, n.s. = non-significant.

Momentary Coping: SSC, support seeking; RC, refusal. Tasks: Com, communication. Coping styles: Emot, seeking emotional support; Focus, focusing and venting emotions. ICC, Intraclass Correlation Coefficient; AIC, Akaike Information Criterion; BIC, Bayesian Information Criterion.

The models fitted indicated the problem-focused coping could be explained by the direct care (*z* = 2.35, *p* < .05) and medication tasks (*z* = 2.47, *p* < .05), by the demands (*z* = 3.58, *p* < .001) and by the acceptation (*z* = - 2.55, *p* < .05) and planning (*z* = 3.48, *p* < .001) coping styles, and by mood (*z* = 4.05, *p* < .001) as a fixed effect. The model for emotion-focused coping indicated that it could be explained by documentation task (*z* = - 2.46, *p* < .05) and medication (*z* = - 3.08, *p* < .01), mood (*z* = 4.37, *p* < .001), demand (*z* = 2.19, *p* < .05), distraction and disengagement coping style (*z* = 2.22, *p* <. 05) as fixed effects, and by demand as a random effect, whereby the effect of demand depends on the person. The final seeking social support coping model indicated that this approach could be explained by the task of communication (*z* = 4.21, p < .001), mood (*z* = 5.50, *p* < .001), fatigue (*z* = - 3.15, *p* < .01) and seeking emotional support as a coping style (*z* = 2.74, *p* < .01) as fixed effects, and by mood as a random effect, whereby the effect of mood depends on the person. Finally, the model fitted indicated that refusal coping could be explained by mood (*z* = 3.59, *p* < .001), and the coping style of focusing and venting emotions (*z* = 2.81, *p* < .01) as fixed effects, and by mood as a random effect, whereby that the effect of mood depends on the person.

## Discussion

### Problem-focused coping

The first hypothesis that was established here postulated that applying a problem-focused coping approach to coping would be chosen by nurses when performing highly demanding direct care or medication tasks, since these are tasks within the nurses' own sphere of action. Therefore, a coping strategy aimed at resolving the problem would be most appropriate and indeed, the results obtained confirm this hypothesis. The second part of the hypothesis was also confirmed, as more use of such strategies were predicted when demands were high. Indeed, it was evident that the more important the problem, the more necessary it was to employ a problem-focused coping strategy. However, as mood acts in a direct way, when mood is negative this coping strategy would be expected to be used more, which is consistent with the fact that high demand is also a predictor of this type of coping. This response may reflect the association of positive mood with the assumption that there is no problem worth coping with. Problem-focused coping is predicted positively by planning coping style and negatively by acceptance coping style, confirming the coherence between the coping style and the momentary measures of coping.

Problem-solving coping and social coping have been associated with less sick leave among female nurses working in hospital environments [74]. Moderate associations were found between the experiences of hospice nurses and planned problem solving or seeking social support [75]. The retention and recruitment of staff who have lower perceived workplace stress and who utilize problem-focused coping approaches may reduce absences due to sickness, and this may be associated with fewer critical incidents and errors [76]. Whilst problem-focused coping approaches were not associated with fatigue, coping through the use of alcohol, venting emotions and avoiding situation were significant predictors of chronic fatigue [77]. Problem-focused coping approaches appear to mediate in the association between emotional intelligence traits and compassion fatigue [78]. In summary, it is not surprising that when faced with highly demanding tasks more directly related to a patient's condition (i.e. direct care and medication), problem-focused coping approaches are the best strategies to choose.

### Emotion-focused to coping

It was hypothesized that the use of emotion-focused coping approaches would not depend on the task but rather, they would be associated with high demands (an effect depending on the individual), little control, negative mood and strong fatigue. The data partially support this hypothesis, as demand and mood predict this type of coping strategy. However, this type of coping does not appear to be related to a lack of control or fatigue, or to tasks like being occupied by documentation and medication tasks, neither of which predict emotion-focused coping approaches. Distraction is also a predictor of emotion-focused coping acceptance strategies, confirming the coherence between the style and the momentary measures of coping.

The preparation and administration of medication is a task that, above all, uses coping strategies focused on the problem, perhaps due to it being a task with much responsibility and for which the consequences of any error may be vitally important for the patient. Alternatively, documentation is a negative predictor of this type of coping, which could be due to the fact that this task does not pose problems that require coping, since it deals with transferring information regarding tasks carried out during the day to a written register. Moreover, the fact that this type of coping is not related to a lack of control, belies the idea that coping focused on the problem and emotions are exclusive alternatives, choosing a shift in emotions when the problem cannot be addressed. These data also support the idea that coping focused on emotions is carried out, irrespective of whether the problem can be addressed or not.

### Seeking social support

A third hypothesis specified that seeking social support coping approaches will be expected to be chosen for tasks that involve other nurses, and those that are highly demanding, with low control, negative mood and high fatigue. The hypothesis is partially confirmed and, certainly, being occupied in a communication task predicts the use of such seeking social support coping strategies, as does a negative mood (although this effect depends on the individual). Demand and control do not appear to be predictors of seeking social support coping strategies and fatigue must be low. That is, the predictors of seeking social support coping strategies are those related to the state of the person and not to the task. These results could be due to the fact that this type of coping can only be done when there are time and people available. Indeed, most of a nurse’s work requires an immediate response and very few of the tasks they perform can be postponed. Furthermore, most tasks are highly protocolized and thus, finding time to share a problem with a co-worker without interruption is not easy. Yang, Liu, Liu, Zhang and Duan (2017) [79] found that the burnout syndrome was negatively associated with self-reported social support. Also, Yu et al. (2014) [48] concluded that social support is a good way to reduce occupational stress in nurses. The time in which this coping strategy would be used would be perceived as leisure time, a time in which the tasks of communication, demand and control cannot appear as predictors and fatigue is low. Seeking emotional support is also a predictor of this type of momentary coping, confirming the coherence between style and momentary measures of coping.

### Refusal coping

The fourth hypothesis specified that refusal coping strategies will not depend on the task, or whether they are associated with high demands, low control, low reward, negative mood or high fatigue. Indeed, no specific task was apparently related to the use of refusal coping strategies, only a negative mood. However, coping style of focusing and venting emotions acts as predictor. It seems that rather than other circumstances, refusal coping depends on a negative mood (an effect that depends on the individual). In addition, refusal coping strategies are those that depend most on the moment and emotion-focused coping strategies depend least on the moment in time, despite their similar role in coping, as indicated by the intraclass correlation coefficients. Thus, and as expected, the choice of the type of momentary coping depends more on momentary factors than on style/personal factors.

Al Barmawi et al. (2019) [80] findings about that higher mean scores on the refusal and seeking social support subscales were associated with lower mean scores on the secondary stress syndrome, and Neff and Germer (2013) [81] showed that a reduction in the level of refusal leads to lower stress levels. Our results are in another direction: the use of this coping strategy depends on the mood. This is not task specific, as other authors have found [82].

## Conclusions

The type of coping most used by nurses when working in acute hospitalization settings is problem-focused coping related to direct care and medication tasks. Emotion-focused coping strategies are often employed in relation to all the tasks undertaken, except those related to medication and documentation, and their use is predicted by mood. Social support is only used when the type of task implies other nurses. In addition, only a negative mood predicts the use of refusal coping strategies. Therefore, two factors must be taken into account in order to understand the coping employed by nurses: the task performed and the nurse's mood. From the point of view of prevention, it would be of interest to improve the coping skills of nurses through cognitive restructuring techniques, self-instruction, problem solving and stress reduction [6, 83]. In this sense, since coping strategies are modifiable factors, it is possible to intervene for their improvement and development through specific individual or group interventions.

The most documented interventions that have demonstrated greater efficacy are workshops or specific training programs for adaptive coping strategies to work stress. These workshops or programs can be based on role-playing techniques that, at present, can be trained through clinical simulation. Other types of interventions that have demonstrated their effectiveness are programs for strengthening social support and promoting social relationships, relaxation techniques (especially Jacobson's progressive muscle relaxation), changing maladaptive or erroneous cognitions and informative sessions or meetings (*briefing*) [84].

For practical purposes, it is worth highlighting the consequences that the use of refusal coping strategies can have when the nurse is not in a good mood during her work shift, such as carelessness, errors in medication, inattention to patients, etc. Training healthcare staff to become aware of their own emotional states in the present moment through mindfulness practice, as well as to detect the emotional state of their colleagues, can facilitate team support and implementation of the skills learned in these prevention programs [85, 86].

One limitation of this study is that the conclusions can only be generalized to ward nurses working in hospitals and accordingly, other tasks performed by different types of nurses may involve the use of different coping strategies. In addition, the classification of the coping strategies that has been used in this work is not the only classification that can be used for coping, which in turn may alter the way that the act of coping itself is seen.

## References

[1] Kakemam E, Raeissi P, Raoofi S, Soltani A, Sokhanvar M, Visentin DC, et al Occupational stress and associated risk factors among nurses: a cross-sectional study. Contemp Nurse. 2019;55: 237–249. 10.1080/10376178.2019.1647791 31334691

[2] Lambert VA, Lambert CE. Nurses workplace stressors and coping strategies. Indian J Palliat Care. 2008;14: 38–44. 10.4103/0973-1075.41934

[3] Martín-Del-Río B, Solanes-Puchol Á, Martínez-Zaragoza F, Benavides-Gil G. Stress in nurses: The 100 top-cited papers published in nursing journals. J Adv Nurs. 2018;74: 1488–1504. 10.1111/jan.13566 29516543

[4] Molina-Praena J, Ramírez-Baena L, Gómez-Urquiza JL, Cañadas GR, De la Fuente EI, Cañadas-De la Fuente, et al Levels of Burnout and Risk Factors in Medical Area Nurses: A Meta-Analytic Study. Int J Environ Res Public Health. 2018;15: 1–16. 10.3390/ijerph15122800 30544672 PMC6313576

[5] Wang W, Kong AWM, Chair SY. Relationship between job stress level and coping strategies used by Hong Kong nurses working in an acute surgical unit. Appl Nurs Res. 2011;24: 238–243. 10.1016/j.apnr.2009.09.003 20974076

[6] Hasan AA. Work stress, coping strategies and levels of depression among nurses working in mental health hospital in Port-Said city. Int Arch Nurs Heal Care. 2017;3: 1–10. 10.23937/2469-5823/1510068

[7] Moustaka Ε, Constantinidis TC. Sources and effects of Work-related stress in nursing. Heal Sci J. 2010;4: 210–216. Available: www.hsj.gr

[8] Barker LM, Nussbaum MA. Fatigue, performance and the work environment: A survey of registered nurses. J Adv Nurs. 2011;67: 1370–1382. 10.1111/j.1365-2648.2010.05597.x 21352271

[9] Caruso CC. Negative impacts of shiftwork and long work hours. Rehabil Nurs. 2014;39: 16–25. 10.1002/rnj.107 23780784 PMC4629843

[10] Fekry Ahmed M, Fathi Sleem W, Hassan Kassem A. Effect of working condition and fatigue on performance of staff nurses at Mansoura University Hospital. IOSR J Nurs Heal Sci. 2015;4: 2320–1940. 10.9790/1959-04358391

[11] Johnston DW, Allan JL, Powell DJH, Jones MC, Farquharson B, Bell C, et al Why does work cause fatigue? A real-time investigation of fatigue, and determinants of fatigue in nurses working 12-hour shifts. Ann Behav Med. 2019;53: 551–562. 10.1093/abm/kay065 30124742

[12] Roelen CAM, van Hoffen, Marieke FA, Waage S, Schaufeli WB, Twisk JWR, et al Psychosocial work environment and mental health‐related long‐term sickness absence among nurses. Int Arch Occup Environ Health. 2018;91: 195–203. 10.1007/s00420-017-1268-1 29032390 PMC5797212

[13] Trousselard M, Dutheil F, Naughton G, Cosserant S, Amadon S, Dualé C, et al Stress among nurses working in emergency, anesthesiology and intensive care units depends on qualification: a Job Demand-Control survey. Int Arch Occup Environ Health. 2016;89: 221–229. 10.1007/s00420-015-1065-7 26112796

[14] Karasek R. Job demands, job decision latitude and mental strain: implications for job redesign. Adm Sci Q. 1979;24: 285–308.

[15] Karasek R. Demand/control model: a social, emotional and physiological approach to stress risk and active behavior development. In: Stellman JM, editor. Encyclopedia of Occupational Health and Safety, International Labour Office. Geneva: OIT; 1998 pp. 34.6–34.14.

[16] Karasek R, Theorell T. Healthy work, stress, productivity, and the reconstruction of working life. New York: BasicBooks; 1991.

[17] Siegrist J. Adverse Health Effects of High-Effort/Low-Reward Conditions. J Occup Health Psychol. 1996;1: 27–41. 10.1037//1076-8998.1.1.27 9547031

[18] Dragano N, Siegrist J, Nyberg ST, Lunau T, Fransson EI, Alfredsson L, et al Effort-Reward Imbalance at Work and Incident Coronary Heart Disease: A Multicohort Study of 90,164 Individuals. Epidemiology. 2017;28: 619–626. 10.1097/EDE.0000000000000666 28570388 PMC5457838

[19] Siegrist J. Effort-reward imbalance at work and cardiovascular diseases. International Journal of Occupational Medicine and Environmental Health. 2010 pp. 279–285. 10.2478/v10001-010-0013-8 20934954

[20] Bakker AB, Killmer CH, Siegrist J, Schaufeli WB. Effort–reward imbalance and burnout among nurses. J Adv Nurs. 2000;31: 884–891. 10.1046/j.1365-2648.2000.01361.x 10759985

[21] Kikuchi Y, Nakaya M, Ikeda M, Narita K, Takeda M, Nishi M. Effort-reward imbalance and depressive state in nurses. Occup Med (Chic Ill). 2009;60: 231–233. 10.1093/occmed/kqp167 19951997

[22] Padilla C, Palmeiro-Silva YK. Effort-reward imbalance and burnout among ICU nursing staff: A cross-sectional study. Nurs Res. 2017;66: 410–416. 10.1097/NNR.0000000000000239 28858150

[23] Schreuder JAH, Roelen CAM, Koopmans PC, Moen BE, Groothoff JW. Effort-reward imbalance is associated with the frequency of sickness absence among female hospital nurses: A cross-sectional study. Int J Nurs Stud. 2010;47: 569–576. 10.1016/j.ijnurstu.2009.10.002 19909954

[24] Bakker AB, Demerouti E. The Job Demands-Resources model: State of the art. J Manag Psychol. 2007;22: 309–328. 10.1108/02683940710733115

[25] Lazarus RS, Folkman S. Transactional theory and research on emotions and coping. Eur J Pers. 1987;1: 141–169.

[26] Suls J, David JP. Coping and Personality: Third Time’s the Charm? J Pers. 1996;64: 993–1005. 10.1111/j.1467-6494.1996.tb00951.x 8956520

[27] Latack JC. Coping with job stress: A conceptual evaluation framework for coping measures. J Organ Behav. 1992;13: 479–508.

[28] Lazarus RS, Folkman S. Stress, appraisal, and coping. New York: Springer; 1984.

[29] Zeidner M, Endler N. Handbook of coping: Theory, research, applications. New York: Wiley & Sons; 1996.

[30] Chang EM, Daly J, Hancock KM, Bidewell JW, Johnson A, Lambert VA, et al The relationships among workplace stressors, coping methods, demographic characteristics and health of Australian nurses. J Prof Nurs. 2006;22: 30–38. 10.1016/j.profnurs.2005.12.002 16459287

[31] Chang EM, Bidewell JW, Huntington AD, Daly J, Johnson A, Wilson H, et al A survey of role stress, coping and health in Australian and New Zealand hospital nurses. Int J Nurs Stud. 2007;44: 1354–1362. 10.1016/j.ijnurstu.2006.06.003 16901488

[32] Deklava L, Circenis K, Millere I. Stress Coping Mechanisms and Professional Burnout among Latvian Nurses. Procedia—Soc Behav Sci. 2014;159: 261–267. 10.1016/j.sbspro.2014.12.369

[33] Lambert VA, Lambert CE, Itano J, Inouye J, Kim S, Kuniviktikul W, et al Cross-cultural comparison of workplace stressors, ways of coping and demographic characteristics as predictors of physical and mental health among hospital nurses in Japan, Thailand, South Korea and the USA (Hawaii). Int J Nurs Stud. 2004;41: 671–684. 10.1016/j.ijnurstu.2004.02.003 15240091

[34] Lambert VA, Lambert CE, Petrini M, Li XM, Zhang YJ. Workplace and personal factors associated with physical and mental health in hospital nurses in China. Nurs Heal Sci. 2007;9: 120–126. 10.1111/j.1442-2018.2007.00316.x 17470186

[35] Lambert VA, Lambert CE, Petrini M, Li XM, Zhang YJ. Predictors of physical and mental health in hospital nurses within the People’s Republic of China. Int Nurs Rev. 2007;54: 85–91. 10.1111/j.1466-7657.2007.00512.x 17305962

[36] Chang EM, Bidewell JW, Huntington AD, Daly J, Johnson A. A survey of role stress, coping and health in Australian and New Zealand hospital nurses. Int J Nurs Stud. 2007;44: 1354–1362. 10.1016/j.ijnurstu.2006.06.003 16901488

[37] Stress Tully A., sources of stress and ways of coping among psychiatric nursing students. J Psychiatr Ment Health Nurs. 2004;11: 43–47. 10.1111/j.1365-2850.2004.00682.x 14723638

[38] Nayomi W. Workplace stress in nursing: a literature review. J Soc Stat. 2016;3: 47–53.

[39] Ceslowitz S. Burnout and coping strategies among hospital staff nurses. J Adv Nurs. 1989;14: 553–557. 10.1111/j.1365-2648.1989.tb01590.x 2768683

[40] Lin H, Probst JC, Hsu YC. Depression among female psychiatric nurses in southern Taiwan: main and moderating effects of job stress, coping behaviour and social support. J Clin Nurs. 2010;19: 2342–2354. 10.1111/j.1365-2702.2010.03216.x 20659207

[41] Schreuder JAH, Roelen CAM, Groothoff JW, Van der Klink JJL, Magerøy N, Pallesen S, et al Coping styles relate to health and work environment of Norwegian and Dutch hospital nurses: A comparative study. Nurs Outlook. 2012;60: 37–43. 10.1016/j.outlook.2011.05.005 21684564

[42] Folkman S. Positive psychological states and coping with severe stress. Soc Sci Med. 1997;45: 1207–1221. 10.1016/s0277-9536(97)00040-3 9381234

[43] Parkes KR. Coping, Negative Affectivity, and the Work Environment: Additive and Interactive Predictors of Mental Health. J Appl Psychol. 1990;75: 399–409. 10.1037/0021-9010.75.4.399 2228890

[44] Ekedahl M. A, Wengström Y. Caritas, spirituality, and religiosity in nurses’ coping. Eur J Cancer Care (Engl). 2010;19: 530–537. 10.1111/j.1365-2354.2009.01089.x 20030696

[45] Carver CS, Connor-Smith J. Personality and Coping. Annu Rev Psychol. 2010;61: 679–704. 10.1146/annurev.psych.093008.100352 19572784

[46] Rothbaum F, Weisz JR, Snyder SS. Changing the world and changing the self: A two-process model of perceived control. J Pers Soc Psychol. 1982;42: 5–37. 10.1037/0022-3514.42.1.5 23861012

[47] Swim JK, Thomas M. Responding to everyday discrimination: A synthesis of research on goal directed, self-regulatory coping behaviors. In: Lavine S, Vanx C, editors. The Claremont Symposium on Applied Social Psychology. 2006 pp. 127–151.

[48] Yu J, Ren X, Wang Q, He L, Wang J, Jin Y, et al The role of social support on occupational stress among hospital nurses. Int J Clin Exp Med. 2014;7: 3000–3004. Available: www.ijcem.com/ 25356174 PMC4211824

[49] Skinner EA, Edge K, Altman J, Sherwood H. Searching for the structure of coping: A review and critique of category systems for classifying ways of coping. Psychol Bull. 2003;129: 216–269. 10.1037/0033-2909.129.2.216 12696840

[50] Stone AA, Schwartz JE, Neale JM, Shiffman S, Marco CA, Hickcox M, et al A comparison of coping assessed by ecological momentary assessment and retrospective recall. J Pers Soc Psychol. 1998;74: 1670–1680. 10.1037//0022-3514.74.6.1670 9654765

[51] Johnston DW, Jones MC, Charles K, McCann SK, McKee L. Stress in Nurses: Stress-Related Affect and Its Determinants Examined Over the Nursing Day. Ann Behav Med. 2013;45: 348–356. 10.1007/s12160-012-9458-2 23355114

[52] Fernández-Castro J, Martínez-Zaragoza F, Rovira T, Edo S, Solanes-Puchol Á, Martín-del-Río B, et al How does emotional exhaustion influence work stress? Relationships between stressor appraisals, hedonic tone, and fatigue in nurses’ daily tasks: A longitudinal cohort study. Int J Nurs Stud. 2017;75: 43–50. 10.1016/j.ijnurstu.2017.07.002 28727992

[53] Bartley CE, Roesch SC. Coping with daily stress: The role of conscientiousness. Pers Individ Dif. 2011;50: 79–83. 10.1016/j.paid.2010.08.027 21076634 PMC2976572

[54] Walker LS, Smith CA, Garber J, Claar RL. Appraisal and Coping with Daily Stressors by Pediatric Patients with Chronic Abdominal Pain. J Pediatr Psychol. 2006;32: 206–216. 10.1093/jpepsy/jsj124 16717138 PMC3150731

[55] Westbrook JI, Ampt A. Design, application and testing of the Work Observation Method by Activity Timing (WOMBAT) to measure clinicians’ patterns of work and communication. Int J Med Inform. 2009;78: S25–S33. 10.1016/j.ijmedinf.2008.09.003 18951838

[56] Patrician PA. Single-Item Graphic Representational Scales. Nurs Res. 2004;53: 347–352. 10.1097/00006199-200409000-00011 15385872

[57] Rovira T, Ferrer I, Edo S, Fernández-Castro J Benavides G, Doval E, et al Validity and feasibility of a nurses’ coping questionnaire for its use in Ecological Momentary Assessment. Eur Heal Psychol. 2016;18: 758.

[58] Carver C, Scheier M, Weintraub J. Assessing Coping Strategies: A Theoretically Based Approach. J Pers Soc Psychol. 1989;56: 257–283. 2926629 10.1037//0022-3514.56.2.267

[59] Crespo M, Cruzado JA. La evaluación del afrontamiento: adaptación española del cuestionario COPE con muestra de estudiantes universitarios. Análisis y Modif Conduct. 1997;23: 797–830.

[60] Kirchner T, Forns M, Muñoz D, Pereda N. Psychometric properties and dimensional structure of the Spanish version of the Coping Responses Inventory—Adult Form. Psicothema. 2008;20: 902–909. 18940101

[61] Carver CS. You want to measure coping but your protocol’s too long: Consider the brief COPE. Int J Behav Med. 1997;4: 92–100. 10.1207/s15327558ijbm0401_6 16250744

[62] Ato M, López JJ, Benavente A. Un sistema de clasificación de los diseños de investigación en Psicología [A classification system of research designs in Psychology]. An Psicol. 2013;29: 1038–1059. 10.6018/analesps.29.3.178511

[63] Fox J, Weisberg S. An {R} Companion to Applied Regression, Second Edition. Thousand Oaks CA: Sage; 2011 Available: http://socserv.socsci.mcmaster.ca/jfox/Books/Companion

[64] Bliese PD, Ployhart RE. Growth modeling using random coefficient models: Model building, testing and illustrations. Organ Res Methods. 2002;5: 362–387.

[65] Bliese P. Multilevel Modeling in R (2.6). An Introd to R Notes R A Program Environ Data Anal Graph. 2016. Available: https://cran.r-project.org/doc/contrib/Bliese_Multilevel.pdf

[66] Grace-Martin K. The Intraclass Correlation Coefficient in Mixed Models. 2017. Available: http://www.theanalysisfactor.com/the-intraclass-correlation-coefficient-in-mixed-models/

[67] Hox JJ. Applied Multilevel Analysis. Amsterdam: TT-Publikaties; 1995.

[68] Akaike H. A new look at the statistical model identification. IEEE Trans Autom Control. 1974;AC-19: 716–723.

[69] Team RC. R: A language and environment for statistical computing. Vienna, Austria: R Foundation for Statistical Computing; 2020.

[70] Bates D, Mächler M, Bolker B, Walker S. Fitting Linear Mixed-Effects Models Using lme4. J Stat Softw. 2015;67: 1–48. 10.18637/jss.v067.i01

[71] Kuznetsova A, Per Bruun Brockhoff PB, Christensen RHB. lmerTest: Tests in Linear Mixed Effects Models. R package version 2.0–32. 2016. Available: https://cran.r-project.org/package = lmerTest

[72] Lüdecke D. sjPlot: Data Visualization for Statistics in Social Science. R package version 2.3.3. 2017.

[73] Wickham H. ggplot2: Elegant graphics for data analysis. 2nd Ed. New York: Springer; 2016.

[74] Schreuder JAH, Plat N, Magerøy N, Moen BE, van der Klink JJL, Groothoff JW, et al Self-rated coping styles and registered sickness absence among nurses working in hospital care: A prospective 1-year cohort study. Int J Nurs Stud. 2011;48: 838–846. 10.1016/j.ijnurstu.2010.12.008 21247577

[75] Harris L. Ways of coping: Understanding workplace stress and coping mechanisms for hospice nurses. ProQuest Dissertations and Theses. 2012.

[76] Burgess L, Irvine F, Wallymahmed A. Personality, stress and coping in intensive care nurses: a descriptive exploratory study. Nurs Crit Care. 2010;15: 129–140. 10.1111/j.1478-5153.2009.00384.x 20500651

[77] Samaha E, Lal S, Samaha N, Wyndham J. Psychological, lifestyle and coping contributors to chronic fatigue in shift-worker nurses. J Adv Nurs. 2007;59: 221–232. 10.1111/j.1365-2648.2007.04338.x 17590207

[78] Zeidner M, Hadar D, Matthews G, Roberts RD. Personal factors related to compassion fatigue in health professionals. Anxiety, Stress Coping. 2013;26: 595–609. 10.1080/10615806.2013.777045 23614527

[79] Yang S, Liu D, Liu H, Zhang J, Duan Z. Relationship of work-family conflict, selfreported social support and job satisfaction to burnout syndrome among medical workers in southwest China: A cross-sectional study. PLoS One. 2017;12: 1–12. 10.1371/journal.pone.0171679 28207821 PMC5312880

[80] Al Barmawi MA, Subih M, Salameh O, Sayyah Yousef Sayyah N, Shoqirat N, Abdel-Azeez Eid Abu Jebbeh R. Coping strategies as moderating factors to compassion fatigue among critical care nurses. Brain Behav. 2019;9: 1–8. 10.1002/brb3.1264 30884198 PMC6456805

[81] Neff KD, Germer CK. A Pilot Study and Randomized Controlled Trial of the Mindful Self-Compassion Program. J Clin Psychol. 2013;69: 28–44. 10.1002/jclp.21923 23070875

[82] Lambert VA, Lambert CE, Ito M. Workplace stressors, ways of coping and demographic characteristics as predictors of physical and mental health of Japanese hospital nurses. Int J Nurs Stud. 2004;41: 85–97. 10.1016/s0020-7489(03)00080-4 14670398

[83] Gellis ZD. Coping with occupational stress in healthcare: A comparison of social workers and nurses. Adm Soc Work. 2002;26: 37–52.

[84] Arrogante O. Estrategias de afrontamiento al estrés laboral en Enfermería [Strategies for coping with work stress in nursing]. Metas de Enfermería. 2016;19: 71–76.

[85] Penque S. Mindfulness to promote nursesʼ well-being. Nurs Manag. 2019;50: 38–44. 10.1097/01.NUMA.0000557621.42684.c4 30985526 PMC6716566

[86] van der Riet P, Levett-Jones T, Aquino-Russell C. The effectiveness of mindfulness meditation for nurses and nursing students: An integrated literature review. Nurse Educ Today. 2018;65: 201–211. 10.1016/j.nedt.2018.03.018 29602138

